# Preschool-located influenza vaccination and influenza-like illness surveillance: an Italian pilot experience

**DOI:** 10.1186/s13052-023-01481-0

**Published:** 2023-07-21

**Authors:** Antonella Amendola, Elisa Borghi, Silvia Bianchi, Maria Gori, Clara Fappani, Lucia Barcellini, Federica Forlanini, Nicolò Garancini, Chiara Nava, Alessandra Mari, Anna Sala, Chiara Gasparini, Emerenziana Ottaviano, Daniela Colzani, Elia Mario Biganzoli, Elisabetta Tanzi, Gian Vincenzo Zuccotti

**Affiliations:** 1 Department of Health Sciences, Università degli Studi di Milano, Milan, 20142 Italy; 2 EpiSoMI CRC-Coordinated Research Centre, Università degli Studi di Milano, Milan, 20133 Italy; 3 Department of Clinical Sciences and Community Health, Università degli Studi di Milano, Milan, 20133 Italy; 4 Department of Pediatrics, V. Buzzi Children’s Hospital, Università degli Studi di Milano, Milan, 20154 Italy; 5 Department of Biomedical and Clinical Sciences, Università degli Studi di Milano, Milan, 20157 Italy

**Keywords:** Children, School-located vaccination, Live-attenuated influenza vaccination, Self-sampling, Saliva, Health equity

## Abstract

**Background:**

We describe the first school-located influenza vaccination campaign with quadrivalent live-attenuated influenza vaccine (LAIV) among pre-school children in Italy, coupled with an innovative school-centred influenza-like illnesses (ILIs) surveillance using a self-sampling non-invasive saliva collection method.

**Methods:**

The pilot study was proposed during the 2021/2022 influenza season to fifteen pre-schools in the Milan municipality. LAIV was offered directly in school to all healthy children without contraindications. ILI differential diagnosis was conducted by real-time RT-PCR for influenza A/B and SARS-CoV-2.

**Results:**

Five pre-schools were involved in the pilot project and overall, 135 families (31.2%) participated in the study, adhering to both surveillance and vaccination; 59% of families had an immigrant background. No pupil experienced adverse reactions after vaccination. Nineteen saliva samples were collected from sixteen children (11.8%). Six samples (31.6%) tested positive for SARS-CoV-2; none was positive for influenza A/B.

**Conclusions:**

The participation in the immunisation campaign was good, considering possible absences due to COVID-19 pandemic, and the intranasal administration was well tolerated and helped to overcome parental hesitancy. Saliva sampling represented a useful tool to reduce children’s stress and increase parents’ compliance. The high participation of families with an immigrant background suggests that school-based interventions can represent an effective strategy to overcome socioeconomic and cultural barriers.

## Background

The ongoing COVID-19 emergency has pointed out the need to reinforce measures to deal with a health crisis and to continue efforts in infectious disease prevention programs. Given COVID-19 overlapping symptoms with influenza-like illness (ILI) [1, 2], influenza immunisation could ease the differential diagnosis and could be pivotal to implement public health strategies by policy makers [3].

Influenza per se represents a major public health problem with important socioeconomic implications. The World Health Organization (WHO) recommends annual vaccination with inactivated or live attenuated vaccines and outlines several risk groups for priority use including pregnant women, children, the elderly, persons with underlying medical conditions, and healthcare workers [4].

The 2020/21 flu season has been characterised by a dramatic reduction in the circulation of influenza viruses, likely due to the control measures put in place for COVID-19 and the limited importation of influenza cases into countries due to travel restrictions and border closures. Similarly, during the latest 2021/22 season, a lower number of influenza cases were reported compared to pre-COVID-19 periods, and fewer viruses were made available for characterization [5]. Nevertheless, with COVID-19 preventive measures and restrictions slackened, we can foresee an increase in influenza transmission and potential co-circulation of influenza viruses and SARS-CoV-2 with an additional burden on health services. In light of these considerations, the WHO Strategic Advisory Group of Experts (SAGE) recommends prioritising at-risk groups for influenza vaccination during the COVID-19 pandemic to ensure optimal influenza control and to reduce treatment in health care facilities that could lead to overload and increase the risk of exposure to SARS-CoV-2 [6].

Despite current data indicating that children are not at increased risk of having severe COVID-19, they remain a priority group for influenza vaccination, particularly young children, because of their risk of severe influenza [7]. Besides direct protection of the paediatric population, the primary spreaders of influenza, mass immunisation can provide indirect protection to the general population, reducing the public health impact. Yearly vaccine coverage is still below optimal rates for children in most countries [8, 9]. Parental hesitancy is one of the main reasons and results from several factors such as the concern about its effectiveness, the yearly booster, the wasted time visiting the provider office for a recommended but not mandatory vaccine, and limited to pandemic years, the risk of SARS-CoV-2 exposure.

Several studies have demonstrated the feasibility and effectiveness of school-located influenza vaccination (SLIV) [10, 11]. Indeed, administering vaccines in school settings is a more efficient way to safely vaccinate large numbers of children. The United Kingdom implemented an immunisation program with live attenuated influenza vaccine (LAIV) nasal spray in primary care for children aged 2 to 3 years [12] and extended the school delivery model to additional year groups [13].

In Italy, the central Authority defines the fundamental goals of the health system, and the Regions are responsible for organising and implementing healthcare services and preventive campaigns. In all the twenty Italian Regions, vaccinations are generally administered in dedicated provider centres by general practitioners and/or paediatricians, and school-located vaccination programmes are not currently set up. Indeed, the last School-Located Vaccination (SLV) program set up in Italy in 1976 was the rubella vaccine campaign offered to prepubescent girls. Influenza campaigns never took place in schools.

Until 2019, influenza vaccination was recommended for all individuals older than 65 years and for those with chronic diseases, regardless of age. Since the 2020/21 influenza season, vaccination was extended to all children aged 6 months to 6 years. To expand the vaccination coverage in the paediatric population, the Lombardy Region introduced for the first time in Italy the quadrivalent live attenuated influenza vaccine, licensed for 2–18 years old subjects, for children aged 2 to 6 years. The Department of Paediatrics of the University of Milan in conjunction with the local Health Authorities set up vaccination weekends in 6 different provider centres covering the metropolitan area of Milan. A total of 9292 children received intranasal vaccination, of whom 7675 were in the 2–6 years age group. Acceptability and safety data were also collected to assess possible barriers and tools that would help in planning the next vaccination campaigns. More than half of informed parents chose to vaccinate their children, and informal communication among parents in the same class group was the most effective tool in promoting the vaccination campaign. The LAIV proved to be non-invasive, simple, and convenient to administer. Most parents stated their intention to vaccinate their child again in the next flu seasons [14].

As for many other health outcomes, disparities in influenza-related hospitalizations and deaths correlate with lower socioeconomic status [15, 16]. Therefore, strategies addressing these swathes of the population should be considered when designing targeted prevention programs such as vaccination campaigns. Moving in this direction, schools represent a unique setting and school-health programs have already proven to be one of the most cost-effective means available to improve both health outcomes and educational achievement [17, 18].

School is also the ideal context for promoting influenza surveillance. Indeed, children with ILI generally do not seek medical care, and not all ILI affected subjects are included in influenza surveillance systems [19]. Total absenteeism has been demonstrated as a validated indicator of possible influenza activity/epidemic [20], but surveillance based only on absenteeism does not allow characterization of the pathogens responsible for outbreaks occurring in schools. On the other hand, virological surveillance allows monitoring influenza viruses circulation and estimating real-time vaccine effectiveness during the influenza season. Leung and colleagues have reported data on virological surveillance conducted using nasopharyngeal swabs, a sampling method difficult to perform in younger children and poorly accepted by parents, which resulted in a reduced caseload that cannot reach a large number of school-age children [21]. Virological surveillance could be enhanced by using oral fluids self-sampling or sampling under parents/legal guardians’ supervision, which might overcome the need of the presence of medical staff within the building to record symptoms and collect samples, allowing virtually reaching all the school population, including pre-school children [22]. Recent studies have reported that saliva swab specimens have high sensitivity and specificity for the detection of influenza and offer a feasible approach for testing ILI in children [23].

In the 2021/22 influenza season, a collaborative network between the University of Milan, Buzzi Children’s Hospital, and the Milan Department of Education started a pilot SLIV experience preschools in Milan (Lombardy Region, Northern Italy), by immunising with nasal-spray LAIV and performing an innovative school-centred ILI surveillance.

This article aims at describing the first school-located influenza vaccination campaign among pre-school children in Italy, and a pilot experience of ILI surveillance by saliva self-sampling, using schools as the connection point between children and parents, and investigators. The purposes of this pilot study were: (1) to identify a suitable model of SLIV in local preschools to be implemented in the future to promote flu immunisation in children aged 2–6 years; (2) to set up a preschool-based screening system to monitor acute respiratory infections, with a particular focus on influenza A/B and SARS-CoV-2.

The experience obtained during this pilot study will serve as a proof-of-concept to establish in the future similar systems that can simultaneously promote vaccination and collect relevant data for ILI epidemiological and virological surveillance and vaccine effectiveness studies.

## Methods

### Context

Lombardy is the most populated region in Italy, counting one-sixth of the Italian population, and according to the Italian National Institute of Statistics [24], it ranked first for the total number of foreign subjects. Milan, its administrative centre, has a population of approximately 1.4 million inhabitants, including over 200,000 foreigners, mainly in the suburbs [25]. Children aged 2–6 years old are about 55,000, of which 27% were children with an immigrant background [24].

#### Enrolment strategy

The study was proposed to 15 preschools in the Milan municipality by setting up informative meetings for local authorities, school principals, and teachers in September 2021, to promote children’s enrolment. The recruitment procedure started at the beginning of October 2021, when pupils’ parents were informed about the study through thematic meetings, information leaflets, posters, and various school tools. Particular attention was paid to the engagement of families with an immigrant background, by involving cultural mediators and by setting up a multi-language informative. Interested parents were asked to contact the study staff for any questions to confirm child’s medical eligibility, and to sign the written informed consent.

Informative materials, the informed consent form, and the protocol received approval by the local Ethical Committee (protocol n. 0049030/2021).

### Vaccination campaign

LAIV (Fluenz Tetra, AstraZeneca) was offered to all healthy children, according to the current European Medicines Agency (EMA) authorization, and upon parent/legal guardian consent. LAIV was offered to all healthy children without any contraindication, and immunisation was carried out in school settings by appropriately trained medical personnel who were part of the research team between November 9th and November 25th, 2021. The vaccination days were conducted directly within the schools, at the end of the classes, or on the weekend, in order to encourage participation. With the collaboration of the teachers, the pupils were led one at a time with their parents into a dedicated room. Here, after obtaining informed consent, and once the suitability of the subject was checked, the vaccination was eventually carried out.

LAIV was administered as a divided dose sprayed into both nostrils. Immunised children were kept under observation for 20 min after vaccination to ensure their safety. Appropriate medical equipment and emergency medications, including epinephrine (1:1,000), were available on-site in the event of an anaphylactic or other immediate allergic reaction.

In all participating schools, a second immunisation day was organised four weeks later for the administration of the second dose of the vaccine for children receiving influenza vaccine for the first time, as recommended.

All families who joined the vaccination campaign were asked to report through a dedicated e-mail address any undesirable events the week after the vaccine administration.

### ILI surveillance

Surveillance and follow-up took place from the middle of December 2021 (approximately 14 days after vaccination) until the end of April 2022. The lack of consent for LAIV was not an exclusion criterion for participating in ILI surveillance programme. Indeed, both vaccinated and unvaccinated children, whose parents/legal guardians signed for participation, could self-sample saliva for differential diagnosis at the onset of ILI symptoms. ILI-related symptoms were sudden acute respiratory syndromes (cough, pharyngitis, nasal congestion) with the onset of fever and/or headache, soreness, chills, sweating, and asthenia.

Lollisponge™ (Copan, Brescia, Italy) is a non-invasive device that can be used to easily self-collect true saliva. The sampling is performed by keeping the sponge stick in the mouth for at least one minute without spitting or biting [26]. Once duly soaked with saliva, the devices can be kept at room temperature for up to 3 days until saliva processing.

At the time of enrollment in the study, three Lollisponge™ devices were provided to each family for saliva collection, together with detailed instructions on the collection procedure and on symptoms to be considered for ILI suspicion, and others were available upon request.

In the study protocol, saliva was delivered by the parents to the child’s preschool and placed in special containers. School principals or teachers activated via telephone a pickup service to collect the sample and deliver it to the diagnostic laboratory by 2 p.m. the same day.

SARS-CoV-2 test was performed within 24 h, and the result was referred according to the regional procedures.

Saliva was recovered by LolliSponge™ centrifugation for 1 min at 500 g. The obtained saliva was then used for SARS-CoV-2 and flu assays, upon treatment with proteinase K and heat inactivation [27]. SARS-CoV-2 detection was carried out by Real Time RT-PCR using COVID-19 HT Screen kit (Clonit, Milan, Italy), CE-IVD for saliva. Influenza A/B viruses presence was determined by the multiplex Real Time RT-PCR kit Flu A + Flu B (Clonit, Milan, Italy).

In addition to saliva collection, parents were instructed to fill an e-form with any information about the respiratory illness, including the presence or not of concurrent systemic symptoms as well as information on the occurrence of pneumonia, new onset or exacerbations of pre-existing cardio-respiratory conditions, hospitalizations, emergency room visits, and non-routine office visits and medication use within 30 days of illness start date. Data, both clinical and virological, were collected by investigators through a dedicated database. A paper questionnaire was also available for families not able to fill out the online form. Parents were also asked to report through the dedicated e-mail address any difficulty in saliva collection.

## Results

### Pilot study participation

**Fig. 1:**
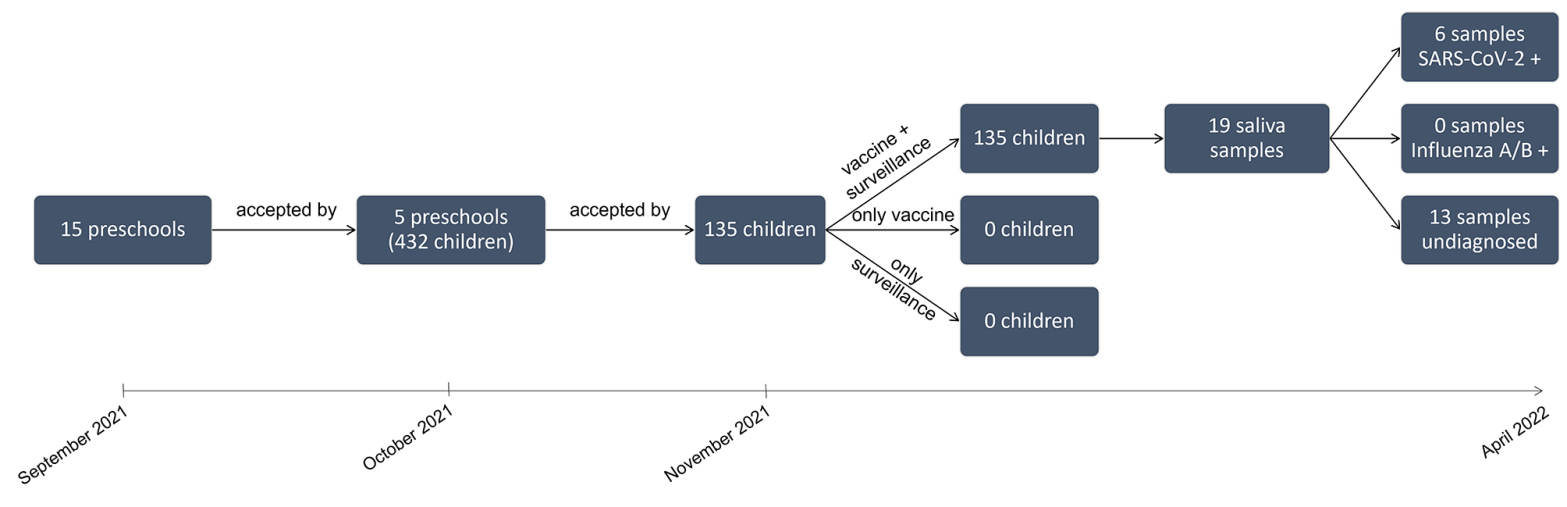
Pilot study flow chart. Vaccination coverage

A total of five public pre-schools were involved in the pilot project. The schools were based in the suburbs of Milan (Fig. 1). The enrolled schools accounted for 432 children aged 2–6 years, 46% of which were children with an immigrant background (i.e., Asian, European, African). Overall, 31.2% of families (135/432) have participated in the study, adhering to both surveillance and vaccination. No families have only participated in the surveillance program (Fig. 1). The percentage of adherence in the various schools ranged from 11 to 49%. The lowest participation percentage was recorded in the last enrolled school.

### Vaccination coverage

**Fig. 2:**
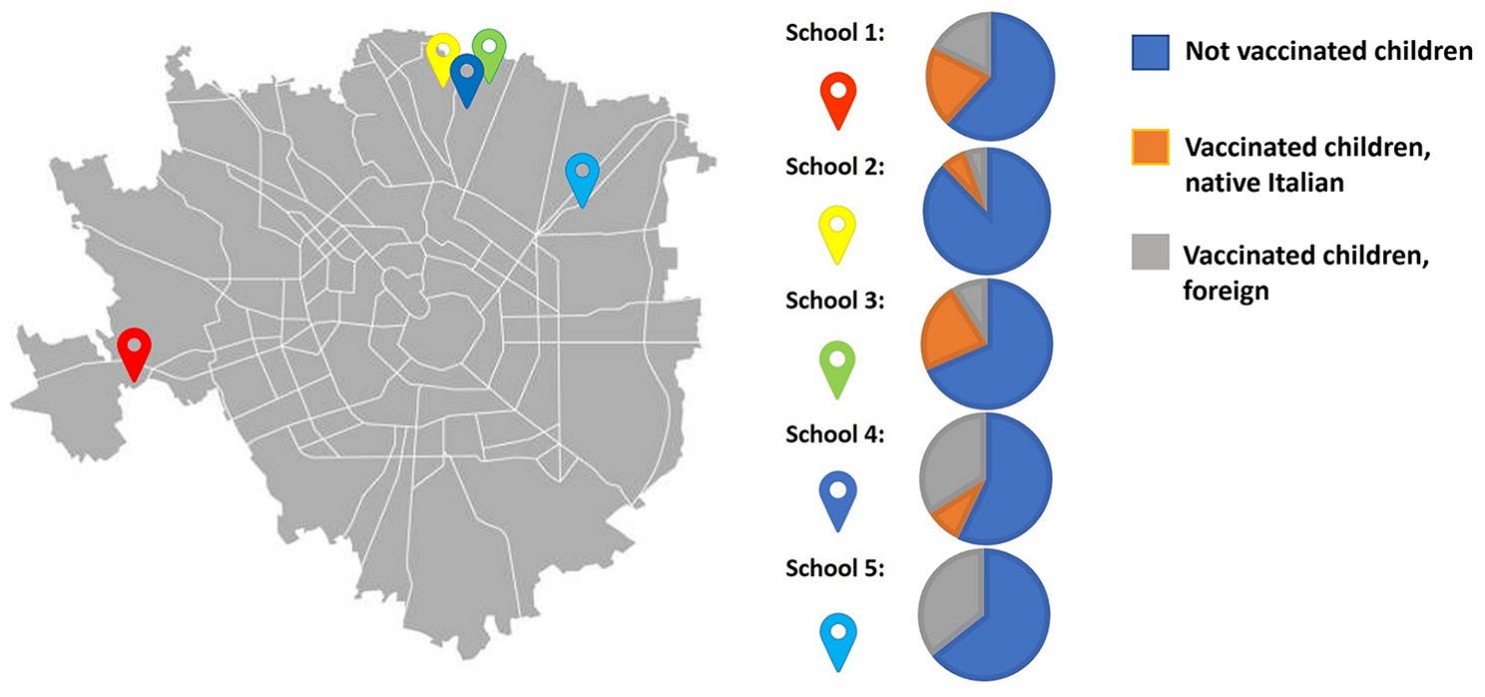
Milan urban area: school locations have been highlighted with coloured pins, and vaccination campaign results are reported as pie-chart for each school.

All families participating in the study (31.2% of families in the 5 schools) authorised their child to be vaccinated. A total of 135 pupils (mean age 5.2 years, range 3–6 years) received vaccination during the campaign; 59% of them were children with an immigrant background (Fig. 2; Table 1), mostly of Asian origin. No pupils experienced adverse reactions during the subsequent observation period.

**Table 1:** Vaccinated children per school, sex, immigrant background and age

School	N. of vaccinated children (male; female)	N. of vaccinated children with an immigrant background (%)	Mean age (range)
School 1	40 (17; 23)	18 (45)	5 (3–6)
School 2	11 (6; 5)	5 (45)	5 (4–6)
School 3	35 (19; 16)	10 (29)	4.9 (3–6)
School 4	23 (12; 11)	19 (83)	5.1 (4–6)
School 5	26 (16; 10)	26 (100)	5.1 (3–6)

### ILI surveillance

No families agreed to participate only in the surveillance study, therefore all children who joined the surveillance (N = 135) were vaccinated.

A total of 405 saliva swabs were distributed to participating families (3 per child).

In the study period, 19 saliva samples from 16 (16/135, 11.8%) children were collected for ILI differential diagnosis. In detail, 3 out of 16 children experienced two ILI episodes, for a total of 19 saliva samples delivered to the diagnostic laboratory.

Overall, the 4.7% (19/405) of the saliva swabs distributed to participating families was used in this study. No families have reported any difficulties in the saliva collection procedure.

All the samples, i.e., saliva quality and quantity, were adequate for molecular testing, and 6/19 (31.6%) resulted positive for SARS-CoV-2. None of these presented with severe symptoms and need for hospitalisation. In detail, 4 positive cases were detected in January 2022, one in February, and one at the beginning of April 2022.

All the samples, despite SARS-CoV-2 positivity, were screened for Influenza A and B viruses. None of the samples collected in the surveillance tested positive for influenza viruses.

Almost all families experienced difficulties in filling in the questionnaire. Indeed, only two families filled the online form, and three the paper questionnaire. Data are not shown due to the few information collected.

## Discussion

Several studies reported that SLV programs were associated with increased influenza vaccine coverage among schoolchildren and reduced influenza transmission within the community. Moreover, for very young children and consequently for their parents, the school represents a familiar and trusted community environment, helping abolish barriers to vaccine access despite the socioeconomic status, and partially overcoming parental hesitancy due to the need of going to provider offices. In addition, influenza vaccination was demonstrated to improve school attendance and children well-being [28].

Pannaraj and colleagues conducted a study that provided laboratory validation of the effectiveness of an SLV program for the first time. Through active ILI surveillance and laboratory-confirmation of influenza, the Authors found that vaccination of at least a quarter of school enrollees reduced the incidence of influenza by over 30% during the 2010/11 season [28].

To date, the vaccination programme described here is the first SLV reintroduced in Italy after almost 50 years. Notably, this is the first time that a SLV programme against influenza has been performed in Italy and a pioneer experience of SLV in pre-school children.

COVID-19 pandemic highly impacted on children’s school attendance, as institutes suffered several shutdowns, with opening/closure rules often revised according to viral circulation [29]. In particular, during the study period and in pre-schools, up to January 2022, classes were forced to close for 10 days after one positive case, then from February-April 2022, after four positive cases [30]. Notwithstanding, the overall participation in the immunisation campaign was good, considering possible absences due to COVID-19 cases in both pupils and parents, ranging from 11 to 42%.

No adverse reactions were reported during the observation period, and thanks to the intranasal administration the procedure was well accepted both by children and parents.

During the 2020/21 influenza season, when the vaccination in Italy was extended to all children aged 6 months to 6 years, a general increase in childhood vaccination coverage was observed compared to the 2019/20 season. Particularly, in Lombardy the average coverage increased from 1.9 to 22.2% in the 2–4 age group and from 1.4 to 15.5% in the 5–8 age group [9]. Our data, although preliminary, seem to suggest higher adherence to vaccination (31.2%) when it is offered in preschools.

Most (about 60%) of the children who participated in the program were children with an immigrant background, often with language difficulties. The high participation of pupils of foreign origin in our campaign suggests that school-based campaigns can represent an effective strategy to reach families with language burdens. Indeed, informative meetings organised by the school with the presence of medical staff and cultural mediators were a crucial moment for rising parents’ engagement through a better understanding of the vaccination procedure and benefits.

Limitations of the study are represented by the low number of enrolled schools (N = 5) and children and the lack of vaccination coverage data for influenza season 2021/22 among preschools in Milan that did not participate in the project. However, taking into account the regional average vaccination coverage, this pilot experience seems to be a strategy that has to be considered to increase the vaccination coverage.

Several improvements can be put in place to maximise participation. First, heterogeneity was observed in terms of participation reported among the five pre-schools included from 11 to 49%. The school with the lowest participation was the last enrolled in the project and the scarce adhesion may be partly explained by the short period between the information meeting and the scheduled vaccination session, which may not have allowed for adequate pass-on among parents who had not been able to attend the meetings. Second, the campaigns were carried out on a single date for each school. The timing, at the end of classes or on the weekend, was decided based on school availability. No preliminary survey has been carried out among parents to check their preference and the presence of a parent/legal guardian was required in order to proceed with the vaccination. Preliminary surveys about parents’ preferences or offering more dates will contribute to the vaccination campaigns’ success.

Moreover, the pandemic period in which the campaign was carried out and the concomitant debate regarding the implementation of the SARS-CoV-2 vaccine among infants and children [31, 32] may as well have affected the participation. In this regard, informative meetings offered a privileged time for a face-to-face discussion between parents and medical staff, and regardless of the final outcome in term of vaccine acceptance, they represented a unique opportunity for health education.

Concerning the ILI surveillance, COVID-19 quarantine rules in preschools have highly hampered the program. Indeed, despite all families (N = 135) who authorised their child to be vaccinated had also adhered to surveillance, only nineteen saliva samples were brought to schools by parents for ILI differential diagnosis, because of the high class-closure rates. We were not able to put in place a follow-up system over the course of the influenza season, with constant communication with families (e.g., through phone calls). Therefore, we are not aware whether children had other ILI episodes that were not reported because of poor parental compliance. Reasonably, we believe that this result is not due to incorrect sample collection, as no parents reported difficulties in the procedure.

However, despite the low number, the saliva-based testing was successful as all parents were able to collect an adequate amount of saliva, without professional assistance. This aspect is highly relevant, as self-sampling at home reduces children’s stress and anxiety and increases parents’ compliance. Saliva collection in young children is challenging as drooling, with or without straw/funnel is often unsuccessful [33] and they have not yet learned to spit. Oral swabbing could result in a scarce saliva amount not sufficient for multiple analyses, and cotton roll-based systems have choking hazard and is not recommended under 6 years of age [34]. The Lollisponge™ device is a lollipop-based sponge, provided with a detailed leaflet for collection procedures, that allows parents to successfully collect saliva at home; indeed it was already used in school settings for SARS-CoV-2 molecular surveillance in compliance with the Ministerial Circular 0043105 of 24 September, 2021 [35]. Saliva is self-preservative and now widely used for SARS-CoV-2 molecular testing. Butler-Laporte and co-workers carried out an exhaustive systematic review and meta-analysis on studies comparing SARS-CoV-2 nucleic acid amplification testing (NAAT) on both nasopharyngeal swab (NPS) and saliva; NAATs displayed similar specificity and sensitivity [33, 36]. Similarly, saliva showed similar performance for influenza viruses [23], resulting in a precious tool for respiratory infection surveillance in the paediatric population and to estimate the real-life effectiveness of LAIV.

Besides ILI surveillance, saliva self-sampling in school could guarantee a prompt SARS-CoV-2 diagnosis, allowing for a reduction of the turn-around time and avoiding the exposure of medical staff to potential infectious risk. More importantly, our pilot experience revealed an even access to testing regardless of socioeconomic status, promoting equity in child health. An equitable access to preventive and diagnostic services is indeed a pivotal challenge of public health [37], especially in the management of epidemic and pandemic emergencies.

None of the collected samples was positive for influenza viruses. Of note, in the 2021/22 season, the influenza virus circulation has been mitigated by masks and social distancing, but a slight increase in activity was observed compared with the 2020/21 season. The epidemic peak was delayed to weeks 12 and 13/2022, in concomitance with COVID-19 preventive measures loosening [5, 38]. As we ended the ILI surveillance at the end of April 2022 (week 17), we may have missed some cases.

## Conclusions

The development of saliva-based virological surveillance could virtually reach the entire school population and, if implemented in the future, it could allow us to conduct studies on the vaccine effectiveness that are lacking in Italy.

On the whole, the pilot project was successfully set up. The intranasal administration was found to be well tolerated, partially helping in overcoming parents’ hesitancy. Our current experience, taking into account both positive results and limitations, could pave the way to a broader school-based vaccine campaign and ILI surveillance that eventually will support widespread access to health preventive measures, increasing vaccine coverage.

Studies involving vaccination and follow-up of children in school settings may be resource-intensive, but the potential public health benefit is great if school-based interventions can reduce influenza-related absenteeism and community-level transmission. Particularly for health care workers involved, efforts were challenging. Therefore, during the 2022/23 influenza season, the project was re-proposed, with a higher involvement of local and health authorities and with the involvement of institutions. When this type of surveillance will be enhanced, monitoring the viral circulation will enable us to estimate the impact of immunisation on reducing laboratory-confirmed influenza cases, complicated forms, and hospitalisation rates of children and their families.

## Data Availability

All data generated or analysed during this study are included in this published article.

## List of abbreviations

EMA: European Medicines Agency
ILI: Influenza-like Illness
LAIV: Live Attenuated Influenza Vaccine
NAAT: nucleic acid amplification testing
NPS: nasopharyngeal swab
SAGE: Strategic Advisory Group of Experts
SLIV: School-Located Influenza Vaccination
SLV: School-Located Vaccination
WHO: World Health Organization
